# What does it take to recruit and retain senior women faculty?

**DOI:** 10.7554/eLife.00615

**Published:** 2013-03-05

**Authors:** Fiona M Watt

**Affiliations:** Centre for Stem Cells and Regenerative Medicine, King's College London, London, United Kingdomfiona.watt@kcl.ac.uk

**Keywords:** Point of view, women in science, careers in science, academic career

## Abstract

eLife deputy editor **Fiona M Watt** recounts some of her personal experiences as a senior female academic in a male-dominated environment.

Back in 2006 I published two articles that summarized the various points that emerged from interviews I had conducted with 24 senior women scientists from different parts of the world (Watt, 2004, 2006a, 2006b). I tried to capture their experiences, both personal and professional, over the previous 30 years, to see what had changed and what remained to be done to provide a level playing field for women and men in our profession. By sharing their personal experiences, my interviewees provided a helpful counter-point to studies that are based on statistical analyses of the under-representation of women in science. While a number of positive conclusions emerged from the interviews, the overall picture was rather disheartening, particularly with regard to covert discrimination, which is also known as passive discrimination or gender stereotyping.

Given the time that has passed since the original interviews, I felt that it might be worthwhile to revisit the issue—this time by sharing my own experiences between 2007 and 2012, while I was working in Cambridge. Those experiences make me firmly of the opinion that covert discrimination, of the sort that my interviewees described, is not going to end any time soon. One of the things that I noticed about Cambridge was that I had fewer female colleagues of equivalent seniority than when I had worked in London. Opinions differ as to what is an achievable/desirable proportion of women in different contexts, whether company boards or scientific institutions. My personal view is that at least 20% representation of women in any scientific forum is both achievable and desirable.

So, are there any issues that are unique to Cambridge? First off, it has to be said that the University is conscious of the low numbers of women academics at the highest levels and has a highly capable, active and committed Gender Equality Champion in Dame Athene Donald, who is Professor of Experimental Physics in the Cavendish Laboratory. I believe, however, that there are cultural issues that hamper the University's ability to hit the right note despite the very best of intentions. For example, having accepted an invitation to attend a meeting of “key people [. . .] to advance this important area of work” (gender equality) I was very surprised that the event consisted of a garden party at which strawberries and sparkling wine were served and the only men present were the Vice-Chancellor, the Disability Champion and the Ethnic Minority Champion. This had the unintended consequence of making me feel that the responsibility for improving the lot of female scientists rests largely with women—a big task, given that so few full professors in the University are female.

During my time in Cambridge, virtually every invitation I received to join a University committee was prefaced by the disclaimer that “we need a woman”. This had the dual effect of making me feel, on the one hand, obliged to accept and, on the other, less empowered to voice an opinion. In case I, or my colleagues, might forget why I was there, the papers for one senior promotions committee had an ‘f' next to my name—not ‘F' for Fiona but ‘f' for female. When I complained, the person who took the blame was a (female) member of the secretarial staff and not the (male) chair of the committee.

Virtually every invitation I received to join a University committee was prefaced by the disclaimer that “we need a woman.”

In my opinion, one of the cultural obstacles to gender equality at Cambridge, and indeed elsewhere, is that senior male academics have protective instincts towards women and are less comfortable with treating them as equals. On several occasions, a senior male colleague explained to me that he had a special insight into the needs of female academics because “I have a daughter”. But I would argue that being a father provides inadequate training in how to deal with women on an equal footing. Our childhood experiences can profoundly influence our perceptions of the roles of men and women in society. I wonder, for example, whether spending one's formative teenage years in a male-only boarding school makes it harder to forge effective working relationships with female colleagues in later life?

While I was working in Cambridge I was involved in several rounds of recruitment of junior group leaders, which were notable not only for a lack of female appointments, but also for the lack of perception that this was a problem. When I raised the issue I frequently received—by way of justification—the response “I can't tell the difference between a man and a woman”. The first time I heard this I had to stifle a giggle as I imagined my colleague's heroism in bagging a mate and procreating. However, my smile faded as this statement was the answer to every question regarding female representation: why are there no female group leaders on the meeting programme? Why are there no women on the short-list? And—hang on—why am I, yet again, the only woman present at this discussion? Being “gender blind” might be a legitimate aspiration for scientists, but in my experience it was a justification for discriminating against women. And what made the situation so dispiriting was that none of the men present during these discussions ever challenged the situation, or asked the same questions as me.

So, what is to be done? While academic institutions may genuinely aspire to increase the number of female professors, their prospects of success are low unless covert discrimination is discussed openly and tackled. Like men, the best women scientists have plenty of career options. If an institution has a reputation as being an uncomfortable environment for women they will tend to avoid applying there. But perhaps ultimately it doesn't matter if some institutions remain predominantly male, as it is those with a healthy representation of women that will flourish.

## References

[bib1] Watt FM (2004). Women in cell science. http://jcs.biologists.org/site/collection/wics.xhtml.

[bib2] Watt FM (2006a). Women in cell biology: getting to the top. Nat Rev Mol Cell Biol.

[bib3] Watt FM (2006b). Women in cell biology: how personal lives shape careers. Nat Rev Mol Cell Biol.

